# On the role of mechanical signals on sprouting angiogenesis through computer modeling approaches

**DOI:** 10.1007/s10237-022-01648-4

**Published:** 2022-11-17

**Authors:** Tamer Abdalrahman, Sara Checa

**Affiliations:** 1 Julius Wolff Institute, Berlin Institute of Health, Charité - Universitätsmedizin Berlin, Berlin, Germany; 2 Mechanobiology Lab., Faculty of Health Sciences, University of Cape Town, Cape Town, South Africa

**Keywords:** Angiogenesis, Mechanics, Mechanobiology, Computational

## Abstract

Sprouting angiogenesis, the formation of new vessels from preexisting vasculature, is an essential process in the regeneration of new tissues as well as in the development of some diseases like cancer. Although early studies identified chemical signaling as the main driver of this process, many recent studies have shown a strong role of mechanical signals in the formation of new capillaries. Different types of mechanical signals (e.g., external forces, cell traction forces, and blood flow-induced shear forces) have been shown to play distinct roles in the process; however, their interplay remains still largely unknown. During the last decades, mathematical and computational modeling approaches have been developed to investigate and better understand the mechanisms behind mechanically driven angiogenesis. In this manuscript, we review computational models of angiogenesis with a focus on models investigating the role of mechanics on the process. Our aim is not to provide a detailed review on model methodology but to describe what we have learnt from these models. We classify models according to the mechanical signals being investigated and describe how models have looked into their role on the angiogenic process. We show that a better understanding of the mechanobiology of the angiogenic process will require the development of computer models that incorporate the interactions between the multiple mechanical signals and their effect on cellular responses, since they all seem to play a key in sprout patterning. In the end, we describe some of the remaining challenges of computational modeling of angiogenesis and discuss potential avenues for future research.

## Introduction

The formation of new capillary blood vessels from preexisting vessels, known as angiogenesis, is an essential process in many physiological and pathological processes. Angiogenesis plays a major role in healthy tissues, e.g., it is involved in embryonic development (Risau 1997), as well as in tissue regeneration (Rouwkema and Khademhosseini 2016) and in common diseases such as cancer (Valastyan and Weinberg 2011).

Angiogenesis is a very complex process that is driven by both mechanical and biochemical signals that operate locally (i.e., at cell–cell and cell-extracellular interfaces) and across distances spanning hundreds of microns (e.g., diffusion of growth factors, the transmission of mechanical signals). New capillary blood vessels are composed of endothelial cells (ECs), and their growth includes endothelial tip cell selection, tip cell motility, and stalk cell proliferation. Tip cells migrate with a large number of long filopodial distensions which can expand, lead, and guide endothelial sprouts. Sprouts join each other forming close to circular structures through anastomosis (Potente et al. 2011; Carmeliet and Jain 2011), which is basic for setting up a bloodstream. New tip cells appear through the branching of existing sprouts, which then form newly formed capillaries.

Angiogenesis is tightly regulated by several microenvironmental factors inside and outside the blood vessel, including soluble molecules (e.g., growth factors and cytokines), extracellular matrices (ECMs), interactions between endothelial cells and other cell types, in addition to mechanical forces originating from ECs themselves, the blood flow, and extravascular tissue deformation (Hudlicka 1998; Ingber 2002; Conway et al. 2001). While full-size strides had been made in the understanding of the role of biochemical signals on the regulation of angiogenesis, the role of mechanical signals has been traditionally less investigated. However, in recent years there is a clear rise of interest in understanding the role of mechanical forces in biological processes in general and angiogenesis in particular, due to strong pieces of evidence showing that the mechanical environment surrounding cells affects many components of their physiology and pathology (Bentley et al. 2008).

Although there are several reviews on computational modeling of angiogenesis (Scianna et al. Sep. 2013; Heck et al. 2015), to the authors' knowledge, no review has focused on models developed to investigate the role played by mechanical signals in the process. The aim of this review is to fill this gap and discuss recent advances in mathematical and computational modeling of mechanoregulation of sprouting angiogenesis. We will first provide an overview of the types of theoretical models which are used to simulate the angiogenic process giving special attention to the modeling tools used to incorporate the mechanical aspects (i.e., external loads, blood flow shear forces, and cell traction forces) during sprouting angiogenesis. Thereafter, we will summarize the types of mechanical signals that have been considered in computational models of angiogenesis and the insights they have provided. Finally, we will discuss current challenges on computational modeling of the effect of mechanical signals on angiogenesis and describe potential future work.

## Mechanical regulation of the angiogenic process

Both biochemical and mechanical signals are known to drive sprout patterning (Czirok and Little 2012; Ceccarelli et al. 2012). Vascular endothelial growth factor (VEGF-A) is a major regulator of blood vessel formation and function (Shibuya 2011). However, many of the individual angiogenic processes are known to be responsive to mechanical stimuli. For instance, key molecular controllers of tip cell selection (Geudens and Gerhardt 2011) have been recently identified as mechanosensitive (Wang et al. 2017; Loerakker et al. 2018). Moreover, the expression and secretion of proteases that are involved in angiogenesis are also mechanosensitive (Dao Thi et al. 2012; Haage et al. 2014).

In vivo, blood vessels are dynamically exposed to mechanical loads that originate from blood flow or the extravascular environment, such as compression by growing tissues or contracting skeletal muscle (Hudlicka 1998). Different studies have been proposed to investigate the effect of different types of loads on sprout formation, for example, by applying tensile loading to skin flaps to promote healing (Cherry et al. 1983) or by providing dynamic mechanical stimulation during the bone healing process (C. von R. Augat Peter, Marianne Hollensteiner 2020). In general, mechanical stimulation has been shown to promote angiogenesis; however, too high mechanical signals have been shown to hinder the process (Lienau et al. 2009).

**Fig. 1:**
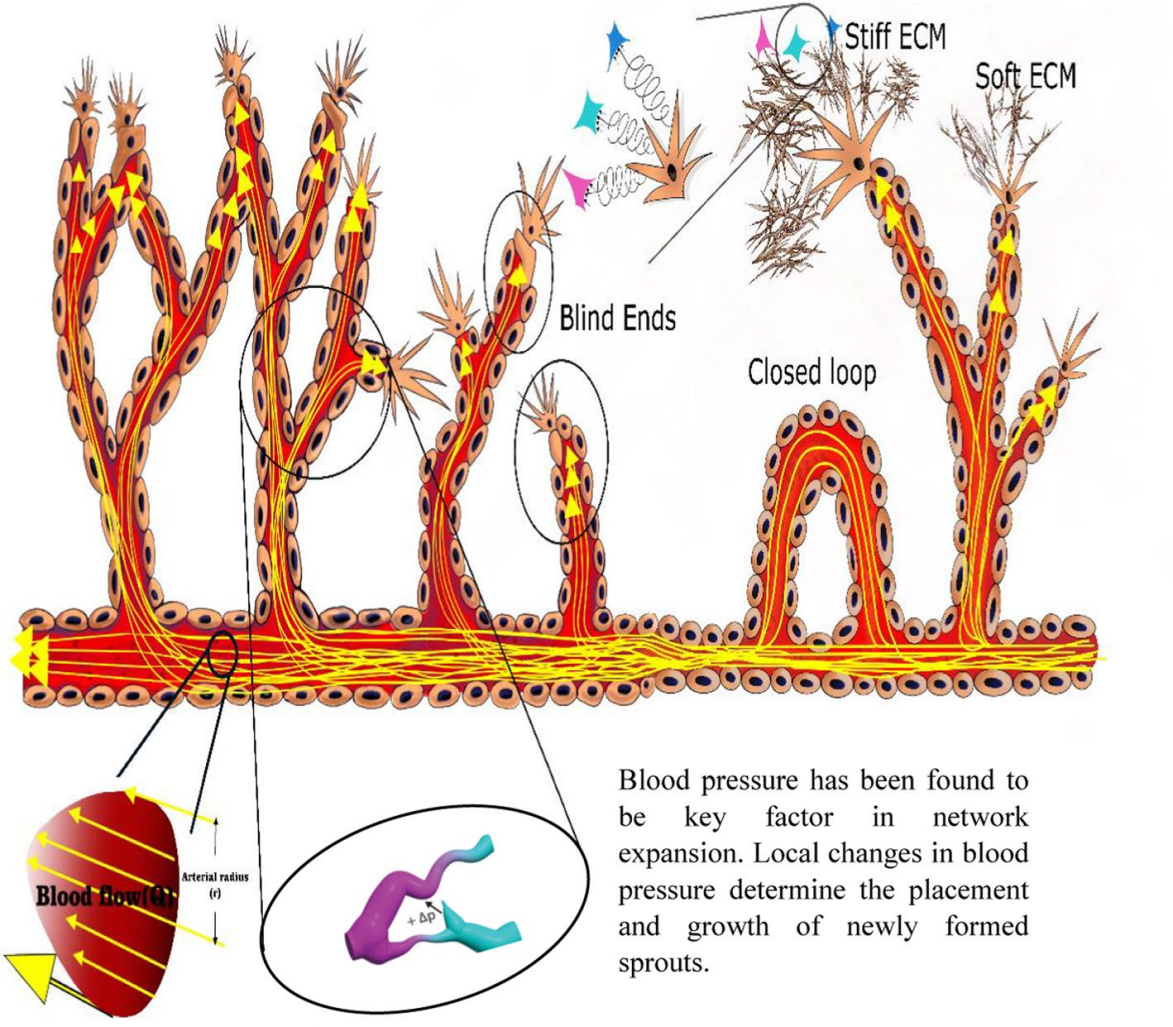
Illustration of mechanically induced sprouting angiogenesis (the vessel structure from Wood et al. 2011) through 1) cell–cell mechanical communication, 2) cell traction forces, 3) ECM deformation, 4) blood flow-induced shear forces, and 5) changes in blood pressure

In vitro, endothelial cells and vascular networks dynamically react to mechanical stimuli, including both flow shear stress and extravascular mechanical strains (Buchanan et al. 2014; Galie et al. 2014; Sharifpanah et al. 2016; Zeiger et al. 2016) (Fig. 1). The mechanical properties of the extracellular matrix have likewise been shown to influence the process of angiogenesis (Mongiat et al. 2016) (Fig. 1). The capability of endothelial cells to construct tubular networks depends on the stiffness of the ECM on which the cells live. In addition, in 2D and 3D angiogenesis assays, increased matrix stiffness resulted in inhibition of vascular network development (Vernon, et al. 1995, 1992; Kuzuya et al. 1996; Kuzuya et al. 1998; Deroanne et al. 2001; Hoying et al. 1996; Kanzawa et al. 1993; Sieminski et al. 2004).

The role of shear forces in angiogenesis has been investigated using 2D and 3D assays where fluid shear forces have been applied to cells. For example, shear stresses at 0.1 Pa promoted the directional assembly of bovine aortic ECs, which formed tubular structures that tended to align with the flow direction on the surface of a Matrigel (Belair et al. 2015). In 3D cultures, the sprouting of a monolayer of pulmonary microvascular ECs into collagen gels was promoted by shear stresses at 0.3 Pa, but there was no directionality exhibited by sprouts, probably because of the minimal shear stress inside the gel (Ueda et al. 2004).

The effect of extravascular mechanical loads on tube formation has been shown utilizing 3D in vitro models in which tensional force was applied to gels using uniaxial stretchers (Korff and Augustin 1999). An EC spheroid cultured on the surface of unstretched collagen gels resulted in sprouts in the radial direction. However, when cultured on deformed collagen gels, outgrowth of ECs specially happened along the direction of the tensional loads (Korff and Augustin 1999). Moreover, the response of endothelial cells to extravascular mechanical strains has been shown to depend on the stimulus magnitude (Krishnan et al. 2013). For instance, Mooney et al. showed that 6% cyclic uniaxial strain increased endothelial cell tube development and angiogenic factor secretion in 2D environments. In 3D culture, 8% strain regulated the directionality of the neovascular network, yet reduced new branch formation (Yu et al. 2009; Matsumoto et al. 2007). Moreover, the direction of growth has also been shown to depend on the strain magnitude with higher magnitudes leading to the orientation of the sprouts perpendicular to the principal strain direction (known as “scape mechanisms”) (Joung et al. 2006).

## Modeling approaches

Computational modeling is a powerful tool to investigate highly complex biological processes, such as sprouting angiogenesis (Carlier et al. 2012; Heck et al. Feb. 2015). In general, theoretical models of angiogenesis can be divided taking into account the modeling approach used. Continuous models are commonly developed using differential equations based on physical laws, while discrete models assemble an accumulation of discrete geometric units that act according to a particular set of rules. Continuous-discrete models, or hybrid models, consolidate the two methodologies, often through determining the behavior of discrete units by solving a problem governed by differential equations.

**Continuous models** simulate the development of tip and stalk cell densities by frameworks of coupled partial differential equations (Byrne and Chaplain 1995; Balding and McElwain 1985; Chaplain and Stuart 1993). Tip cell behavior is often simulated via a reaction–advection–diffusion equation where often the gradient of an angiogenic factor is the parameter that controls the chemotactic migration of the cells. The advancement of stalk cells or vessel cell densities is usually driven by a term relative to the flux of tip cells, a phenomenon termed the “snail-trail” (Byrne and Chaplain 1995). Phase-field continuum models have also been used to investigate sprouting angiogenesis (Santos-Oliveira et al. 2015). In these models, partial differential equations are used to describe the movement of boundaries between domains making them suitable to model morphology and growth of biological systems. Using this approach, Santos-Oliveira et al. were able to describe sprouting as a function of the mechanical characteristics of the microenvironment (Santos-Oliveira et al. 2015).

Generally, in the context of angiogenesis modeling, continuum models ignore constituent details (for example, cell-level details), and new capillary growth is modeled as changes in vascular density at the network level (Czirok and Little 2012). Continuum models are typically implemented by solving differential equation systems that describe physical phenomena as a continuous spread in space and/or time. The main advantage of this modeling approach is that it allows modeling large regions of interest without high computational cost.

**Discrete computer models** aim at modelling the ramified vasculature that results from the angiogenic process. Discrete models are better for studying behavior at the level of individual cells because they allow for a more comprehensive prediction of the capillary network's structure and morphology, which is not achievable with continuous models. These models address the behavior of one or more individual cells as they interact with one another and the microenvironment. Discrete approaches usually track and update individual cells as per different biophysical rules (Kiani and Hudetz 1991; Landini and Misson 1993; Gazit et al. 1995; Baish et al. 1996; Nekka et al. 1996), simulating the dynamics of the process as increments of time and space (Chaplain 1995; Stokes et al. 1991). *One disadvantage of discrete modeling is the high computational cost associated with the simulation of the cellular behavior of each individual cell*.

Several researchers have developed computer models of angiogenesis based on a **hybrid approach** (Bookholt et al. Dec. 2016; Vilanova et al. 2014), which are characterized by a combination of discrete and continuum models. Hybrid models have been used to model microscale cell behavior based on signals from macroscale fields (Bonilla et al. 2014). Hybrid models can also involve cell dynamics to model blood flow (Perfahl et al. 2011; Stéphanou and S. Le Floc’h, and A. Chauvière, 2015; Owen et al. 2009) and drug delivery in the vascular network (Vilanova et al. 2014) as well as tumor growth (Perfahl et al. 2011). Recently, **multiscale approaches** have been built where biological phenomena at different lengths and time scales have been coupled to investigate their contribution to the angiogenic process (Stéphanou and S. Le Floc’h, and A. Chauvière, 2015; Alarcón et al. 2003, 2004). *Although discrete and hybrid models provide a qualitative description of vessel network shape, they are both theoretically and computationally expensive*.

Many computer models of angiogenesis have investigated the role of mechanical signals in the process. To this aim, the level of mechanical signals influencing endothelial cell behavior needs to be predicted. **Finite element modeling** has been used to estimate the deformation of the extracellular matrix created by endothelial cell traction forces and external loads (Edgar et al. 2014; Edgar et al. 2015a). In addition, **computational fluid dynamics (CFD)** has been used to estimate blood flow velocity and wall shear stress, as mechanical stimulators of angiogenesis (Bernabeu et al. 2014). In addition, **mathematical models** have been used to quantify cell–cell mechanical communication and interactions of the cell with the surrounding extracellular matrix (Daub and Merks 2013). A classification of some of the most relevant models based on their approaches is given in Table 1.

**Table 1:** Summary of computer models of angiogenesis investigating the role of mechanical signals on the process

Model types	Mechanical regulator	Model space	Environment	Model description	Aim	Conclusion
Continuum models						
Santos-Oliveira et al. (2015)	Cell traction force	2D	ECM	A phase-field model of angiogenesis describes vessel sprouting as a function of mechanical factors of the microenvironment. It has three partial deferential equations to: Distinguish between capillaries and the extracellular matrix Describe the displacement field around the tip cell Obtain VEGF consumption rate	To predict sprout morphology as a function of the elastic properties of tissues and cell traction forces	Tip cells create a tension on nearby stalk cells. This tension then produces strain and empty spaces, triggering cell proliferation
Macklin et al.(2009)	Blood flow	2D	ECM	A continuum model of solid tumor invasion combined with a developed continuum model of tumor-induced angiogenesis. The angiogenesis model accounts for cell–cell, cell–ECM adhesion, and ECM degradation	To develop a new multi-scale model of vascular solid tumor growth *that is able to demonstrate the significance of the link between vascular network development and remodeling, blood flow through the network, and tumor progression*	The hydrostatic stress generated by tumor cells and extracellular matrix degradation affects network remodeling
Discrete models						
Checa and Prendergast (2010)	External load	3D	ECM	A discrete model combined with a finite element model. The finite element model was used to quantify mechanical strains in the extracellular matrix while the discrete model was used to predict sprouting morphology	To investigate the effect of cell seeding on vascular network development and tissue growth inside a regular-structured bone scaffold under different loading conditions	Increasing the number of seeded cells might reduce the rate of vascularization and the maximal penetration of the vascular network. High levels of loading inhibited capillary growth
Owen et al. (2009)	Blood flow	2D	ECM	The model takes into account diffusion through the tissue (oxygen and VEGF) as well as subcellular and cell-scale phenomena, all while coupling everything with blood flow.	*To develop the first multiscale model of vascular tissue growth that combines blood flow, vascular remodeling, and the subcellular and tissue scale dynamics of multiple cell populations*	Simulations show that vessel pruning, due to low wall shear stress, is highly sensitive to the pressure drop across a vascular network
Hybrid models						
Edgars et al. (2014)	ECM properties and boundary conditions	3D	ECM	A discrete growth model of angiogenesis coupled with a finite element solver. ECM fibril orientation, matrix density, and nodal displacement were interpolated to the microscale using the shape functions in the mesh of the FE model	To develop a computational model to simulate angiogenic growth coupled to matrix deformation	The model was able to predict the effects of mechanical forces on the orientation of newly developing microvessels and their interaction with the extracellular matrix
Edgars et al. (2015a)	Cell traction force	3D	ECM	The same model was used by Edgars in (2014)	To develop a continuous-discrete modeling approach to simulate dynamic mechanical interactions between growing neovessels and the deformation of the matrix considering different boundary conditions	During simulations of each gel mechanical boundary condition, the model accurately predicted gel contraction and microvessel alignment
Edgars et al. (2015b)	ECM properties	3D	ECM	The same model was used by Edgars in (2014)	To investigate angiogenic growth within a heterogeneous environment	AngioFE has the ability to model angiogenesis in a range of mechanical environments are presented in this study: homogeneous density, discrete density heterogeneity (narrow gap), continuous density heterogeneity (density gradient), and applied load/unloading (preconditioning)
Stéphanou A et al. (2015)	Cell traction force	2D	Elastic matrix of fibers	A hybrid continuous-discrete model where cell displacement was modeled as a function of traction force and substrate elasticity. In addition, the model takes into account three types of proteins; growth factors, matrix fibers, and degrading enzymes	To develop a hybrid continuous-discrete model to investigate the migration of individual contracting cells on an elastic matrix composed of fibers	The intensity of the cell traction force, as well as the rigidity of the matrix, have a substantial impact on cell migration and angiogenesis. First, the vascular network can only be retrieved for a limited range of traction force strengths. Second, matrix rigidity is important, but only in a narrow range that is consistent with the underlying biological activity
Guilkey et al. (2006)	External load	3D	ECM	The material point method (MPM) was used to model vascularized constructs	To develop a computer model to simulate the 3D mechanics of a vascularized scaffold under tension, consisting of growing microvascular fragments embedded in a collagen gel	The usefulness of a modified material point method algorithm for large-scale simulation of the mechanics of cellular constructs was established in this study. Stress localization and channeling were caused by the existence of microvessels in the collagen construct
Edgars et al. (2013)	ECM anisotropy	2D	ECM	The same model was used by Edgars in (2014)	To develop a capillary growth model and to demonstrate its ability to describe the changes in microvessel growth resulting from ECM anisotropy and imposed boundary conditions	The simulation framework delivered a precise depiction of microvascular length, branching, and orientation metrics over time for both isotropic and anisotropic ECs
Daube and Merk (2013)	ECM properties	2D	ECM	By iteratively expanding and contracting the domains based on a set of cell behavior rules, a cell-based modeling technique was used to simulate stochastic cell motility Extracellular matrix materials, proteolytic enzymes, and diffusing growth factors were all described using partial differential equations	To investigate endothelial cells and extracellular matrix interactions and their role on the cellular organization	The model describes cell–matrix interactions on the level of individual cells. The vessels grow fastest at intermediate ECM densities but no sprouts occur at low densities, and the vasculature grows at a slower rate at extremely high densities
Bauer et al. (2007)	ECM fiber density	2D	ECM	Endothelial cell migration, growth, division, cellular adhesion, and the evolving structure of the stroma are all described by a cellular Potts model, which is based on system-energy reduction. Endothelial cells are assumed to migrate to promote stronger adhesive bonds over weaker adhesive bonds, shorter cell boundaries over longer cell boundaries, and toward areas with higher chemical concentrations	To gain a better understanding of the biochemical and biomechanical signals that drive angiogenic processes in tumors	Anisotropy of the matrix fibers and the composition of the stroma are important factors leading to capillary sprout branching and anastomosis
McDougall et al. (2006)	Blood flow	2D		A hybrid model that assumes that tip cells migrate via (i) random motility, (ii) chemotaxis in response to tumor angiogenic factors (TAF) gradients, and (iii) haptotaxis in response to fibronectin gradients in the ECM. The vessel adaptation and capillary remodeling by blood flow are considered	To investigate the impact of blood perfusion during angiogenesis	The blood flow in the model has an immediate effect during capillary growth, with spiral transformations and organization redesigning happening as immediate consequences of primary anastomoses
Gödde and Kurz (2001)	Blood Flow	2D		Based on geometric and biophysical initial boundary conditions, a C + + computer program was created Geometry was defined on a two-dimensional isometric grid, as well as elementary bifurcations that could proliferate or regress in response to random and deterministic processes The pressure, flow, and velocity distributions in the network were defined using the nodal-admittance-matrix method while accounting for hemodynamic peculiarities such as the Fahraeus–Lindqvist effect and exchange with extravascular tissue	To present a new computer model for simulating microvascular growth and remodeling in arteries and veins that mimics angiogenesis and blood flow in real vascular plexuses	The model is the first hemodynamic angiogenesis model to generate arteriovenous interdigitating vascular patterns with known hemodynamic and transport properties at each vascular branch

## Computational modeling of the role of mechanical signals on angiogenesis

### Computer models of cell traction force-driven angiogenesis

Cell-produced traction forces influence migration, proliferation, and differentiation of many cell phenotypes, including endothelial cells and pericytes that participate in angiogenesis (Joung et al. 2006; McCormick, et al. 2002). Mechanical signals transferred into cells by means of mechanotransduction are controlled by the structure and arrangement of the ECM (Deroanne et al. 2001; Vernon and Sage 1999) and by cell receptor structures bound to ECM components (Jalali et al. 2001).

Santos-Oliveira et al. (2015) developed a phase-field continuous model of sprouting angiogenesis able to describe the sprout growth as a function of cell traction forces generated by the sprout tip cell and cell–cell adhesion forces. Using this model, the authors were able to investigate the regulation of endothelial cell proliferation based on local stresses. The model presented how different types of endothelial cell proliferation regulation influence the shape of the growing sprout.

**Fig. 2:**
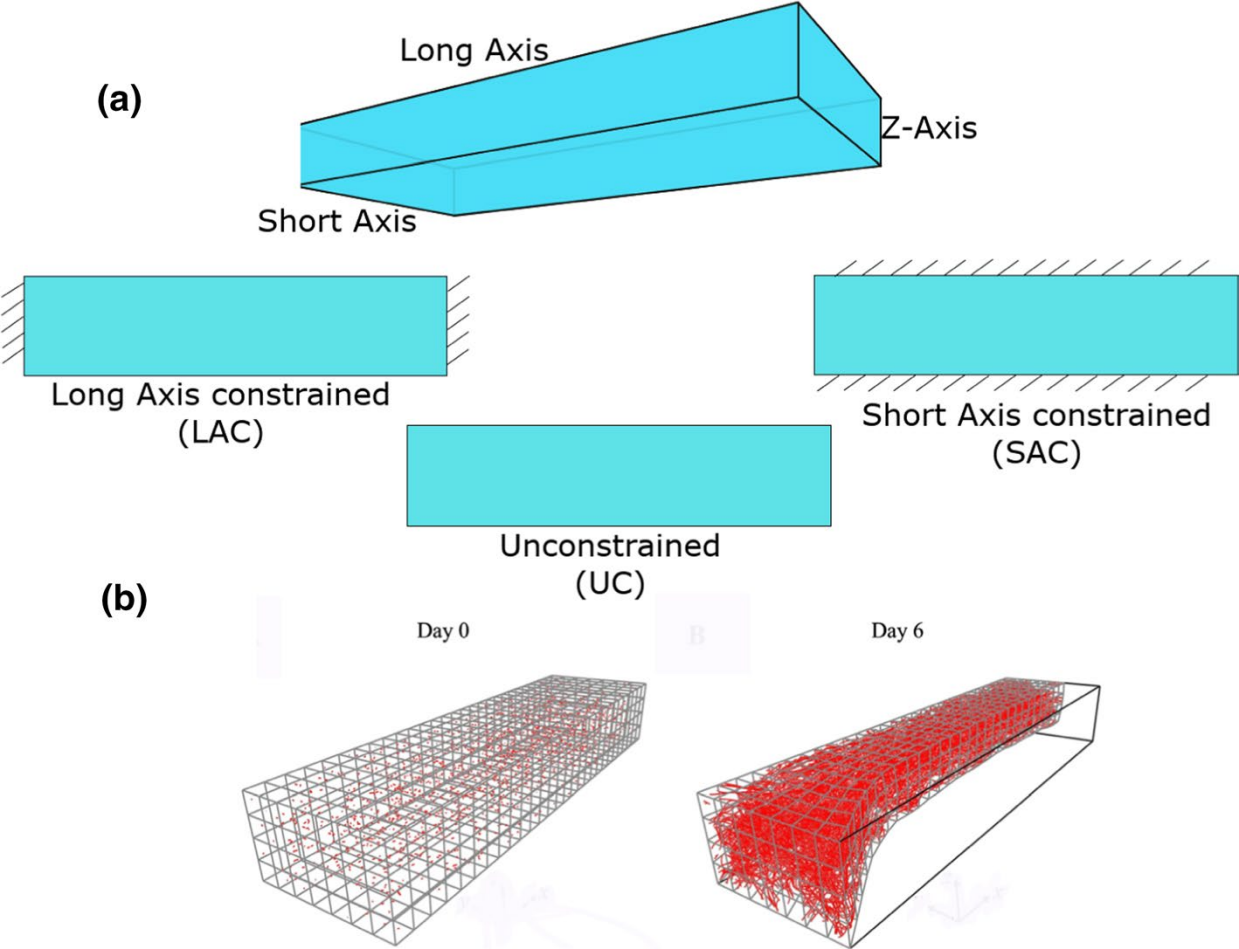
**A** Boundary constraints utilized during the modeling of angiogenic microvessel fragments in three dimensions. **B** A long-axis constrained (LAC) vascularized gel simulation. The mesh was seeded with initial microvessel fragments on Day 0. The gel was highly perfused by microvessels on Day 6 and had deformed into a “neck” shape (adapted from (Edgar et al. 2015a))

Using a continuous-discrete modeling approach, Edgars et al. (2015a) estimated the value of deformation around tip cells and investigated its effect on angiogenesis formation. Here, the nonlinear finite element (FE) was used to calculate the ECM deformation generated by cell traction forces, while the deformation field was then used to update the position of tip cells. The coupling of the discrete angiogenic growth model to the finite element model allowed the model to track information at the microscale while resolving deformation at the macroscale. Individual neovessel sprouts produced localized stress fields that moved as the matrix deformed and neovessels grew. Both the vascular components and the ECM field were updated at the end of each time step using the mechanics information predicted by the finite element model (displacing and re-orienting sprouts, re-orienting collagen fibrils, and updating density). This established a dynamic link between cellular biomechanical activity and the angiogenesis process. Assuming that ECs have a tendency to align with the direction of the parent vessel and the collagen fibril orientation, they were able to show that the ability of tip cells to read the local mechanical environment by applying traction forces could be a potential mechanism dictating sprout patterning. The model was then used to predict vascular alignment under three different boundary conditions: unconstrained (UNC), long-axis constraint (LAC), and short-axis constraint (SAC). Within the UNC and SAC simulations, the model predicted random alignment while aligned microvasculature was predicted when simulating the LAC constructs (see Fig. 2).

Taking into account cell traction forces, the role of the ECM properties on the regulation of sprouting angiogenesis has also been investigated. Stephanou et al. investigated the role of cellular traction forces and ECM viscoelastic properties on sprouting angiogenesis using a 2D hybrid continuous-discrete model (Stéphanou and S. Le Floc’h, and A. Chauvière, 2015). Using this model, the authors proposed potential effects of cellular traction forces and matrix rigidity on cell migration and sprout formation. They showed that there is a limited range of traction force intensities for which a vascular network can be obtained. They showed that when the traction force increases, the density of the vascular network decreases, and the capillaries become more rectilinear. In addition, no networks were predicted if the traction force was too high. They also investigated the influence of matrix rigidity (Young's modulus) on the vascular network, where they showed that only high values of Young’s moduli influence vessel patterning.

### Computer model of extracellular matrix property-driven angiogenesis

The ECM plays a key role in the coordination of cellular migration during the angiogenic process (Lamalice et al. 2007). Different theoretical models of angiogenesis have integrated the mechanical interaction between ECs and the ECM.

**Fig. 3:**
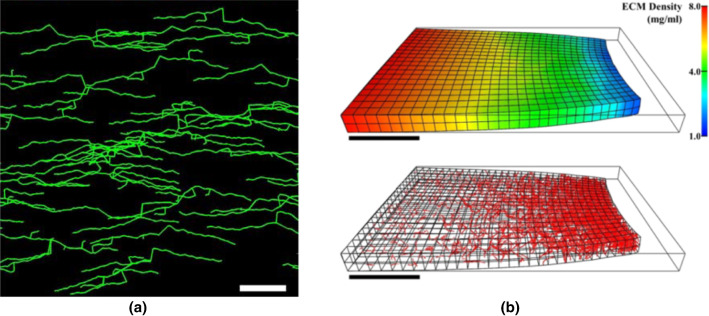
**a** Angiogenesis model with an anisotropic ECM predominately aligned along the constrained axis (Edgar et al. 2013). **b** Angiogenesis simulated within a gradient ECM density field (adapted from (Edgar et al. 2015b))

Edgar et al. (2013) presented a simulation framework to investigate the mechanisms behind microvessel patterning in fibril collagen matrices. They investigated the dynamical interactions between microvessel length and branching with the matrix fiber orientation of anisotropic ECMs. They predicted vessel morphology based on a linear combination of collagen fibers orientation, the vessel density gradient, and a random walk component. In addition, they included the remodeling of the matrix caused by active stresses generated by tip cells. They found that microvessels preferentially aligned along the constrained axis (fixed-edge) and that angiogenesis within a randomly oriented ECM produced microvessels with no preferential alignment (Fig. 3a).

Edgar et al. further extended the model presented in Edgar et al. (2013) to investigate the role of ECM density in the transmission of mechanical signals and sprouting angiogenesis. The authors investigated three different microenvironments; homogeneous, a narrow gap of low-density collagen surrounded by regions of higher density collagen, and a matrix with density gradients. For the homogenous case, the microvessels were predicted to randomly grow, but for the other cases, the vessels were able to grow more in the areas with lower density (Edgar et al. 2015b) (Fig. 3b).

Daub and Merks (2013) presented a cell-based model to investigate the potential role of ECM-guided cell migration in angiogenesis. This model gives a description of cell–matrix interactions on the scale of a single cell, where the cellular velocity and movement direction are guided by the local concentration of ECM and the local gradient. They show that higher degree of branching with bigger vascular sprouts was obtained for faster ECM degradation. Bauer et al. (2007) presented a tumor angiogenesis model based on a cell-centric approach to study the effect of matrix on neovessel branching and anastomosis. In that model, the architecture of the ECM was assumed anisotropic, with regions of varying densities so that the mesh-like anisotropic structure of the ECM was utilized. The mechanical interactions between cells and the ECM during endothelial cell migration were obtained as a function of the compression resistance of the matrix fiber composite (ECM structure). The model suggested that heterogeneity of the structure of the matrix is necessary for the branching formation where the cells split from the main branch is enabled by inhomogeneity of the matrix. According to their findings, the anisotropic structure of matrix fibers has a significant impact on the direction and shape of migrating capillary sprouts. The model also showed that tissue cell resistance and endothelium cell attachment to matrix fibers during endothelial cell migration, both alone and in combination, are sufficient to promote branching and anastomosis.

Bauer et al. (2009) extended their model (Bauer et al. 2007) to investigate the effect of ECM properties (fiber orientation and matrix density) on vascular morphogenesis and focused on mechanisms controlling cell shape and orientation, sprout extension speeds, and sprout morphology. The model suggests density-dependent pro- and anti-angiogenic effects and that high matrix fiber anisotropy provides strong contact guiding cues, being a mechanism for sprout branching initiation. Finally, by investigating sprout formation on modified matrix patterns, the model showed compelling evidence that contact guidance modulates cell orientation. Their model found a strong correspondence between fiber alignment and cell shape and orientation where it showed that cells elongated in the direction of the matrix fibers. In general, this model showed that matrix topology alone is enough to regulate cell shape and orientation and to initiate sprout branching.

### Computer modeling of the effect of external forces on sprouting angiogenesis

External loading has been implicated as an important regulator of ECM deformation effects on angiogenesis. The role of external mechanical forces and boundary conditions on angiogenesis has been investigated by several computational studies.

Edgar et al. (2014) presented a computational model to investigate the effect of boundary conditions and externally applied loads on vascular growth and alignment. In a computational model of collagen matrix deformation, the neovessel alignment and morphology depended on the strain direction, which corresponded with collagen fibril orientation. In general, the microvessels used the local fiber orientation to determine the direction of growth, and vessels grew along the constraint direction. The distribution of vessel orientation angle shows that vessels in these simulations strongly resemble vessels in long-axis constrained experiments.

**Fig. 4:**
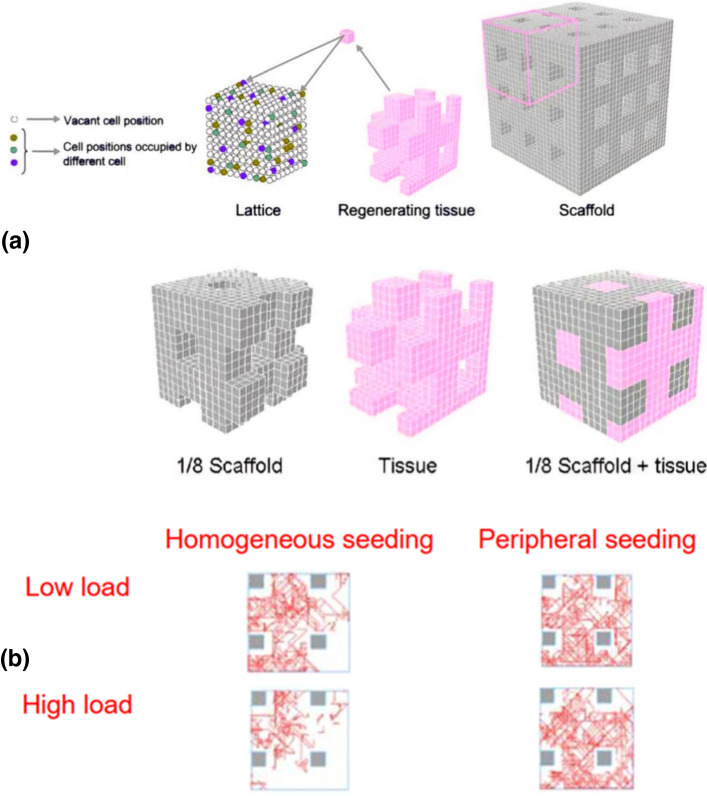
**a** Finite element model of a bone scaffolds where individual elements contain lattice points for the simulation of cell activity **b** predicted vessel formation inside the scaffold pores (pink area) as a function of initial stem cell seeding (adapted from (Checa and Prendergast Mar. 2010))

Checa and Prendergast, (2010) investigated the influence of tissue mechanical strains induced by external loading in sprouting angiogenesis during bone regeneration within tissue engineering scaffolds. Here, the authors utilized an agent-based model to simulate the vascular and tissue growth inside regular porous scaffolds under different loading conditions. The local mechanical environment surrounding the cells was determined using a finite element model of a regular-structured bone scaffold. The scaffold's interior (pores) was divided into a regular grid (lattice), with each position (lattice point) representing a potential space for a cell to occupy (Fig. 4).

The model showed an influence of initial cell seeding on vascular growth within the scaffold pores, where peripheral seeding was predicted to be more beneficial compared to uniform seeding. Moreover, reduced loading was predicted to lead to increased vascularization and bone tissue formation (Fig. 4).

Another technique called the meshless method, or the material point method (MPM), has been used by Guilkey et al. (2006) to simulate the 3D mechanics of a vascularized scaffold under tension. In this model, stacks of confocal microscopic images were utilized and converted into 3D discrete particles which represent the complex network. Using global mechanical properties of the structure, this model was able to show the stress distribution in the ECM. The findings revealed a very inhomogeneous stress distribution, with microvessels being subjected to significantly more stress than the surrounding collagen. Even under uniaxial tensile loading, this supports the concept that local stresses around cellular constructs in a 3D matrix are inhomogeneous.

### Computer models of blood flow-driven sprouting angiogenesis

Blood flow leads to hemodynamic forces and the resulting shear forces and pressure on ECs affect their function and differentiation (Fig. 1). Several computer models have been developed to investigate different aspects of the interaction between shear stresses and sprouting angiogenesis (Nekka et al. 1996; Gödde and Kurz 2001; McDougall et al. 2002; Rolland et al. 1999; Sandau and Kurz 1994). A computational fluid dynamic approach was recently employed by Stapor et al. (Stapor et al. Jun. 2011) to evaluate the shear stress distribution within a blind-ended sprout. The computational results provide an initial estimate of shear stress magnitudes and highlight the importance of using comparable methods to estimate local shear stress distributions caused by transmural or interstitial flows across endothelial cells. They reported that endothelial cells at the sprout entry suffer higher shear stress during the early phases of capillary sprouting while shear forces are minimal within the capillary sprout. Shear pressures, on the other hand, become relevant for longer sprouts at later phases of sprout growth.

Godde and Kurz (2001) evaluated the local shear stress by simulating the blood flow through capillary networks to estimate the local pressure gradient. A probability function was then computed based on the local shear stress and predefined minimum and maximum values of shear stress that would trigger either growth or regression of a microvessel. Their model predicted that interdigitating arteriovenous patterning between the terminal branches of arterial and venous trees is shear stress dependent but not pressure-dependent.

In 2006, McDougall et al. presented a computer model called “dynamic adaptive angiogenesis.” This model simulated multi-scale phenomena affecting angiogenesis, including the *link between cell migration and blood flow* (McDougall et al. 2006). The simulation of individual endothelial cell movement was combined with a continuum theory to determine blood flow as indicated by Poiseuille’s law and vessel adaptations in response to shear stress, pressure, and a metabolic mechanism. They were able to show that the sensitivity of ECs to blood wall shear stresses is a major determinant of vascular topology. The cell migration and flow models are linked in this model by incorporating the mechanism of shear-dependent vessel branching. The authors simulated network architectures that adapt dynamically in areas of the capillary network experiencing increased shear stresses by using adjuvant vessel branching. The vessel branching process is made more sensitive to wall shear stress (WSS) by lowering the branching requirements in terms of maximum WSS. The model predicted that at the apex of the formed loop, there is an increase in branching activity. When a double loop of dilated capillaries forms, it is quickly followed by a burst of vessel branching and an increase in local vessel density all around the loop.

In order to simulate sprout network morphology and blood flow, a method is needed to track changes in both properties during the sprouting process. Macklin et al. (Macklin et al. 2009) updated the vasculature hybrid model (Levine et al. 2002; Zheng et al. 2005) to simulate the dynamics of angiogenesis, allowing for accurate prediction of blood flow and vascular remodeling due to shear and mechanical stresses generated. This model was able to consider the adhesion of cell–cell and cell–matrix interactions and ECM degradation. Moreover, Wu et al. (Wu et al. 2013) further extended the model (Macklin et al. 2009; Wu et al. 2013) to include the interstitial fluid pressure and interstitial fluid flow.

Owen et al. (Owen et al. 2009) proposed a 2D multiscale vascular growth model based on the model built by Alarcon et al. (2003, 2004). Owen’s model combines blood flow, angiogenesis, vascular remodeling, and tissue scale dynamics of multiple cell populations as well as the subcellular dynamics (including the cell cycle) of individual cells. The authors aimed to include the mechanisms of vascular development by taking into account the pruning of vessels that have insufficient flow. According to the predictions, vessel pruning is mostly influenced by the pressure drop across the vascular network, with a smaller drop resulting in more pruning*. The model also showed how that the initial vascular architecture can influence the final density, especially when the pressure drop across the network is high enough to allow for low levels of vessel pruning.*

Recently, Bazmara et al. (2015) presented a multiscale model of angiogenesis that includes molecular (intracellular), cellular, and tissue scales. In this model, *the formation of a closed loop, blood flow in the loop, and loop survival after blood flow is simulated.* Additionally, the cell phenotype alteration due to blood flow is considered in the model at the intracellular scale. This model predicted that when blood flow is incorporated into the loop, the anastomosed sprouts stabilize and elongate. When the loop is modified without taking blood flow into account, the loop collapses.

## Remaining challenges in computational modeling of angiogenesis

Although great advances have been made in the field of computer modeling of angiogenesis, specifically concerning the role of mechanical signals in the process, many questions remain. In what follows we discuss some of the remaining challenges in the field:

### Integration of mechanical and biological signals

Most of the computer models of sprouting angiogenesis developed so far have focused either on the role played by biological or mechanical signals on the process (Title, et al. 2013; Sato 2013). Very few models (Vilanova et al. 2014; Manoussaki 2003; Nivlouei et al. 2021; Vega et al. 2020) have been used to investigate the interaction between these two types of stimuli and their relative role on the process.

Mechanistic models that can describe the behavior (e.g., migration and proliferation) of individual cells have the potential to evaluate the relative role of interacting mechanisms such as haptotaxis/haptokinesis and chemotaxis. One of the most important aspects which need to be further investigated is the role of the tip cell as the main driver for sprouting angiogenesis. On the one hand, the tip cell attaches to the matrix and uses the filopodia to sense the mechanical and chemical factors during the angiogenesis growth, and the cell exerts a contractile pulling force (traction force) to migrate. On the other hand, the stalk cells proliferate, possibly pushing tip cells forward. The interaction between these two processes and how they are regulated by the interaction of mechanical and chemical signals remains largely unknown.

### Integration of multiple scales

Multiscale connectivity is an evident challenge when simulating a highly hierarchical process such as angiogenesis, where processes at the cellular scale (eg. tip cell migration) are regulated by subcellular and tissue level cues (Stepanova et al. Jan. 2021). Agent-based (ABM) or cell-based models are a promising platform in which discrete cells can be simulated to behave autonomously depending on a set of rules, which can represent events at different scales. This approach is well suited for linking different models at multiple scales because each set of rules can be based on biological experiments or outputs from embedded computational models at different scales running either in parallel or iteratively.

### Model complexity, assumptions, and simplification strategies

Angiogenesis is a multiscale, extremely complex process that involves a sequence of biological events that result in neovascularization. It is vital to create computer models that are as near to reality as feasible while still being solvable. As a result, it is critical to establish some assumptions about the system and think about simplification techniques. These simplifications can be connected to geometric simplifications, process simplifications, and temporal simplifications. Assuming a two-dimensional geometry for a three-dimensional tissue structure by setting the model boundaries to replicate a cross-sectional slice across the tissue is one sort of geometric simplification (Machado et al. Apr. 2011). Another geometric simplification strategy is to abstract a microvascular network complex branching pattern into a more regular pattern that has some of the complex network characteristics (e.g., vessel length density) but does not simulate the network exact structure on a vessel-by-vessel basis (Milde et al. 2008; Guerra et al. 2020). Consolidating the number of growth factors in a model that affects a specific process by focusing on a few essential components or assigning critical behaviors to one growth factor is an example of a process simplification method (Schugart et al. Feb. 2008). The modeling of discrete timeframes is a commonly used temporal simplification (Mac Gabhann et al. 2007; Gevertz and Torquato Dec. 2006; Peirce et al. 2004). The level of simplification and on which property depends on the specific research questions to be addressed.

### Parameter identification

Another challenge in modeling sprouting angiogenesis is the identification of appropriate input model parameters. Usually, these parameters are obtained from (or estimated from) published experimental data. Some parameters, however, cannot be measured in vivo using currently available experimental techniques. Instead, sometimes in vitro models are used to derive model parameters, which might not fully represent the in vivo conditions (Staton et al. 2009). To evaluate the impact that specific model parameters might have on model predictions, parameter sensitivity analysis is of great importance. Here the levels of certain parameters are adjusted systematically with the aim of quantitatively determining the effect that specific parameters have on the model predictions (Bauer et al. 2007).

### Model validation and verification

Model verification is the process that confirms that the model is well implemented and the model results match what is expected based on the model inputs. In contrast, model validation is required toward approving a model before it can be used as a predictive tool. In model validation, it is proven that the model makes predictions that are in agreement with experimental data.

In the case of computer models of angiogenesis, models are often not built to make predictions about what would happen in “in silico experiments.” Computer models of angiogenesis are, however, used to try to understand the mechanisms behind experimental observations. In this case, validation of the model is not so important but the comparison of the model with experimental data is used to make a hypothesis about the underlying mechanisms. Many of the existing models have been developed to simulate controlled in vitro experiments with the aim of gaining a deeper understanding of the mechanical signals playing a role under those controlled conditions (Santos-Oliveira et al. 2015; Peirce et al. 2004; Roman and Pekkan 2012; Boas et al. 2013; Shiu et al. 2005). In these studies, model geometry and input parameters are derived from the dedicated in vitro experiments and computer model predictions of sprout patterning (e.g., vessel density, vessel orientation, etc.) are compared to the experimental data (Edgar et al. 2015b, 2013; Bauer et al. Jul. 2009). For example, Edgar et al. (Edgar et al. 2015a) used a dedicated in vitro experimental setup to investigate mechanical interactions between growing neovessels and the deformation of the matrix. They compared computer model predictions with experimental data of gel contraction and microvessel alignment for different boundary conditions. The authors refer to this as a global validation of the model, since individual model assumptions or hypothesis could not be tested. Bazmara et al. (Bazmara et al. Jun. 2015) did not use dedicated experiments for model validation, but used an in vivo and an vitro experiment reported in the literature to validate different aspects of the model, in particular, predicted sprout extension before loop formation (in vitro validation) and loop elongation after establishment of flow (in vivo validation). Although they were able to show good quantitative agreement between model predictions and experimental data, they only validated one specific parameter (extension speed), not testing the validity of the multiple assumptions/hypothesis made in the model. To the authors knowledge a fully validated computer model of angiogenesis does not exist, partially due to the complexity of the process.

### Not an isolated process

One of the main challenges in understanding the angiogenic process, both experimentally and theoretically, is that it is often not an isolated process. Endothelial cells interact with many other cells in the organism both mechanically and chemically, for example, with stromal and immune cells (Ribatti and Crivellato Sep. 2009; Stockmann et al. 2014; Hughes May 2008). Understanding the regulation of the process requires therefore to consider other cell types that might play a role in this regulation.

### Heterogeneity of endothelial cells

Although computer models usually talk about endothelial cells in general, experimentally it has been shown that different subtypes of endothelial cells exist not only in different organs but also within the same vascular bed. For example, two subpopulations of endothelial cells have been identified in the murine skeletal system: the H type, which are responsible for angiogenesis, and the L type, which form the sinusoidal capillaries in bones (Kusumbe et al. 2014). This heterogeneity must be considered since different subpopulations have different functionality and a different response to external signals.

## Future directions

Sprouting angiogenesis is a complex process that is guided by different chemical and mechanical cues. Although different mechanical cues and their role on sprouting angiogenesis have been investigated, there are still some mechanics-related effects unexplored, e.g., the details of mechanotransduction at the cellular level. Moreover, most models have focused on the effect of individual mechanical cues, for example blood flow; however, all the above-described mechanical cues act at the same time during the angiogenic process. The role of outer versus inner vascular mechanical signals, for example, remains highly unexplored.

It is critical to precisely analyze the specific nature of the forces at play in order to design and implement in silico that address physiologically relevant topics in mechanobiology. This includes the force's direction (isotropic or anisotropic for topography, axial or radial for flow or strain), and the ECM properties (isotropic, anisotropic, and fibers orientations).

Additionally, when applicable, the force's time-dependent pattern (waveform and frequency of ECM’s strain, pressure of blood flow) should be considered in order to obtain a dynamic mechanoregulation model for sprouting angiogenesis. To better comprehend the effect of various mechanical cues on ECs and to model the mechanisms by which ECs integrate and decode multiple environmental information, more research is needed. In vivo, these mechanical cues are linked and imposed on ECs at the same time, and therefore, the impact of many cues should be included in the same computational model to better replicate how ECs integrate and interpret various mechanical inputs.

## Conclusions

The impact of mechanical signals on the angiogenic process remains only partially understood. Computational models of angiogenesis have brought us a deeper understanding of the role of mechanical signals on the process since they provide a way to quantify those signals, which are often difficult to measure experimentally. This review article summarizes the application of computer models to investigate the role of different mechanical cues (cellular traction forces, the surrounding extracellular matrix, external loads, and blood flow-induced shear forces) on the regulation of angiogenesis.

We show that most of the models are focused on understanding the role played by one single mechanical signal (e.g., fluid flow) on the angiogenic process. However, multiple mechanical signals of different origins act simultaneously, affecting sprout patterning. Because of the complex interaction between cellular processes, ECM remodeling, and extra- and intravascular mechanical forces involved in sprouting angiogenesis, computer models should be developed considering multiple origins of the involved mechanical signals.

## References

[CR1] Alarcón T, Byrne HM, Maini PK (2003). A cellular automaton model for tumour growth in inhomogeneous environment. J Theor Biol.

[CR2] Alarcón T, Byrne HM, Maini PK (2004). A mathematical model of the effects of hypoxia on the cell-cycle of normal and cancer cells. J Theor Biol.

[CR3] Baish JW, Gazit Y, Berk DA, Nozue M, Baxter LT, Jain RK (1996). Role of tumor vascular architecture in nutrient and drug delivery: an invasion percolation-based network model. Microvasc Res.

[CR4] Balding D, McElwain DLS (1985). A mathematical model of tumour-induced capillary growth. J Theor Biol.

[CR5] Bauer AL, Jackson TL, Jiang Y (2007). A cell-based model exhibiting branching and anastomosis during tumor-induced angiogenesis. Biophys J.

[CR6] Bauer AL, Jackson TL, Jiang Y (2009). Topography of extracellular matrix mediates vascular morphogenesis and migration speeds in angiogenesis. PLoS Comput Biol.

[CR7] Bazmara H, Soltani M, Sefidgar M, Bazargan M, Naeenian MM, Rahmim A (2015). The vital role of blood flow-induced proliferation and migration in capillary network formation in a multiscale model of angiogenesis. PLoS One.

[CR8] Bentley K, Gerhardt H, Bates PA (2008). Agent-based simulation of notch-mediated tip cell selection in angiogenic sprout initialisation. J Theor Biol.

[CR111] Belair David G., Whisler Jordan A., Valdez Jorge, Velazquez Jeremy, Molenda James A., Vickerman Vernella, Lewis Rachel, Daigh Christine, Hansen Tyler D., Mann David A., Thomson James A., Griffith Linda G., Kamm Roger D., Schwartz Michael P., Murphy William L. (2015). Human vascular tissue models formed from human induced pluripotent stem cell derived endothelial cells. Stem Cell Rev Rep.

[CR9] Bernabeu MO (2014). Computer simulations reveal complex distribution of haemodynamic forces in a mouse retina model of angiogenesis. J R Soc Interface.

[CR10] Boas SEM, Palm MM, Koolwijk P, Merks RMH, Reinhart-King CA (2013). Computational modeling of angiogenesis: towards a multi-scale understanding of cell–cell and cell–matrix interactions. Mechanical and chemical signaling in angiogenesis.

[CR11] Bonilla LL, Capasso V, Alvaro M, Carretero M (2014). Hybrid modeling of tumor-induced angiogenesis. Phys Rev E.

[CR12] Bookholt FD, Monsuur HN, Gibbs S, Vermolen FJ (2016). Mathematical modelling of angiogenesis using continuous cell-based models. Biomech Model Mechanobiol.

[CR13] Buchanan CF, Verbridge SS, Vlachos PP, Rylander MN (2014). Flow shear stress regulates endothelial barrier function and expression of angiogenic factors in a 3D microfluidic tumor vascular model. Cell Adhes Migr.

[CR14] Byrne HM, Chaplain MAJ (1995). Mathematical models for tumour angiogenesis: numerical simulations and nonlinear wave solutions. Bull Math Biol.

[CR15] Carlier A, Geris L, Bentley K, Carmeliet G, Carmeliet P, van Oosterwyck H (2012). MOSAIC: a multiscale model of osteogenesis and sprouting angiogenesis with lateral inhibition of endothelial cells. PLoS Comput Biol.

[CR16] Carmeliet P, Jain RK (2011). Molecular mechanisms and clinical applications of angiogenesis. Nature.

[CR17] Ceccarelli J, Cheng A, Putnam AJ (2012). Mechanical strain controls endothelial patterning during angiogenic sprouting. Cell Mol Bioeng.

[CR18] Chaplain MAJ (1995). The mathematical modelling of tumour angiogenesis and invasion. Acta Biotheor.

[CR19] Chaplain MAJ, Stuart AM (1993). A model mechanism for the chemotactic response of endothelial cells to tumour angiogenesis factor. Math Med Biol.

[CR20] Checa S, Prendergast PJ (2010). Effect of cell seeding and mechanical loading on vascularization and tissue formation inside a scaffold: a mechano-biological model using a lattice approach to simulate cell activity. J Biomech.

[CR21] Cherry GW, Austad E, Pasyk K, McClatchey K, Rohrich RJ (1983). Increased survival and vascularity of random-pattern skin flaps elevated in controlled, expanded skin. Plast Reconstr Surg.

[CR22] Conway EM, Collen D, Carmeliet P (2001). Molecular mechanisms of blood vessel growth. Cardiovasc Res.

[CR23] Czirok A, Little CD (2012). Pattern formation during vasculogenesis. Birth Defects Res Part C - Embryo Today: Rev.

[CR24] Daub JT, Merks RMH (2013). A cell-based model of extracellular-matrix-guided endothelial cell migration during angiogenesis. Bull Math Biol.

[CR25] Deroanne CF, Lapiere CM, Nusgens BV (2001). In vitro tubulogenesis of endothelial cells by relaxation of the coupling extracellular matrix-cytoskeleton. Cardiovasc Res.

[CR26] Edgar LT, Sibole SC, Underwood CJ, Guilkey JE, Weiss JA (2013). A computational model of in vitro angiogenesis based on extracellular matrix fibre orientation. Comput Methods Biomech Biomed Engin.

[CR27] Edgar LT (2014). Mechanical interaction of angiogenic microvessels with the extracellular matrix. J Biomech Eng.

[CR28] Edgar LT, Maas SA, Guilkey JE, Weiss JA (2015). A coupled model of neovessel growth and matrix mechanics describes and predicts angiogenesis in vitro. Biomech Model Mechanobiol.

[CR29] Edgar LT, Hoying JB, Weiss JA (2015). In silico investigation of angiogenesis with growth and stress generation coupled to local extracellular matrix density. Ann Biomed Eng.

[CR30] Gabhann FM, Ji JW, Popel AS (2007). Multi-scale computational models of pro-angiogenic treatments in peripheral arterial disease. Ann Biomed Eng.

[CR31] Galie PA, Nguyen DHT, Choi CK, Cohen DM, Janmey PA, Chen CS (2014). Fluid shear stress threshold regulates angiogenic sprouting. Proc Natl Acad Sci USA.

[CR32] Gazit Y, Berk DA, Leunig M, Baxter LT, Jain RK (1995). Scale-invariant behavior and vascular network formation in normal and tumor tissue. Phys Rev Lett.

[CR33] Geudens I, Gerhardt O (2011). Coordinating cell behaviour during blood vessel formation. Development.

[CR34] Gevertz JL, Torquato S (2006). Modeling the effects of vasculature evolution on early brain tumor growth. J Theor Biol.

[CR35] Gödde R, Kurz H (2001). Structural and biophysical simulation of angiogenesis and vascular remodeling. Dev Dyn.

[CR36] Guerra A, Belinha J, Mangir N, MacNeil S, Jorge RN (2020). Sprouting angiogenesis: a numerical approach with experimental validation. Ann Biomed Eng.

[CR37] Guilkey JE, Hoying JB, Weiss JA (2006). Computational modeling of multicellular constructs with the material point method. J Biomech.

[CR38] Haage A, Nam DH, Ge X, Schneider IC (2014). Matrix metalloproteinase-14 is a mechanically regulated activator of secreted MMPs and invasion. Biochem Biophys Res Commun.

[CR39] Heck TAM, Vaeyens MM, Van Oosterwyck H (2015). Computational models of sprouting angiogenesis and cell migration: towards multiscale mechanochemical models of angiogenesis. Math Model Nat Phenom.

[CR40] Heck TAM, Vaeyens MM, Van Oosterwyck H (2015). Computational models of sprouting angiogenesis and cell migration: towards multiscale mechanochemical models of angiogenesis. Math Model Nat Phenom.

[CR41] Hoying JB, Boswell CA, Williams SK (1996). Angiogenic potential of microvessel fragments established in three- dimensional collagen gels. Vitr Cell Dev Biol - Anim.

[CR42] Hudlicka O (1998). Is physiological angiogenesis in skeletal muscle regulated by changes in microcirculation?. Microcirculation.

[CR43] Hughes CCW (2008). Endothelial-stromal interactions in angiogenesis. Curr Opin Hematol.

[CR44] Ingber DE (2002). Mechanical signaling and the cellular response to extracellular matrix in angiogenesis and cardiovascular physiology. Circ Res.

[CR45] Jalali S (2001). Integrin-mediated mechanotransduction requires dynamic interaction with specific extracellular matrix (ECM) ligands. Proc Natl Acad Sci U S A.

[CR46] Joung IS, Iwamoto MN, Shiu YT, Quam CT (2006). Cyclic strain modulates tubulogenesis of endothelial cells in a 3D tissue culture model. Microvasc Res.

[CR48] Kanzawa S, Endo H, Shioya N (1993). Improved in vitro angiogenesis model by collagen density reduction and the use of type III collagen. Ann Plast Surg.

[CR49] Kiani MF, Hudetz AG (1991). Computer simulation of growth of anastomosing microvascular networks. J Theor Biol.

[CR50] Korff T, Augustin HG (1999). Tensional forces in fibrillar extracellular matrices control directional capillary sprouting. J Cell Sci.

[CR51] Krishnan L, Chang CC, Nunes SS, Williams SK, Weiss JA, Hoying JB (2013). Manipulating the microvasculature and its microenvironment. Crit Rev Biomed Eng.

[CR52] Kusumbe AP, Ramasamy SK, Adams RH (2014). Coupling of angiogenesis and osteogenesis by a specific vessel subtype in bone. Nature.

[CR53] Kuzuya M, Satake S, Miura H, Hayashi T, Iguchi A (1996). Inhibition of endothelial cell differentiation on a glycosylated reconstituted basement membrane complex. Exp Cell Res.

[CR54] Kuzuya M (1998). Inhibition of angiogenesis on glycated collagen lattices. Diabetologia.

[CR55] Lamalice L, Le Boeuf F, Huot J (2007). Endothelial cell migration during angiogenesis. Circ Res.

[CR56] Landini G, Misson G (1993). Simulation of corneal neovascularization by inverted diffusion limited aggregation. Investig Ophthalmol Vis Sci.

[CR57] Levine HA, Pamuk S, Sleeman BD, Nilsen-Hamilton M (2002). Mathematical modelling of tumour angiogenesis and the action of angiostatin as a protease inhibitor. J Theor Med.

[CR58] Lienau J (2009). Differential regulation of blood vessel formation between standard and delayed bone healing. J Orthop Res.

[CR59] Loerakker S, Stassen OMJA, ter Huurne FM, Boareto M, Bouten CVC, Sahlgren CM (2018). Mechanosensitivity of Jagged-Notch signaling can induce a switch-type behavior in vascular homeostasis. Proc Natl Acad Sci USA.

[CR60] Machado MJC, Watson MG, Devlin AH, Chaplain MAJ, Mcdougall SR, Mitchell CA (2011). Dynamics of angiogenesis during wound healing: a coupled in vivo and in silico study. Microcirculation.

[CR61] Macklin P, McDougall S, Anderson ARA, Chaplain MAJ, Cristini V, Lowengrub J (2009). Multiscale modelling and nonlinear simulation of vascular tumour growth. J Math Biol.

[CR62] Manoussaki D (2003). A mechanochemical model of angiogenesis and vasculogenesis. ESAIM Math Modell Numer Anal.

[CR63] Matsumoto T, Yung YC, Fischbach C, Kong HJ, Nakaoka R, Mooney DJ (2007). Mechanical strain regulates endothelial cell patterning in vitro. Tissue Eng.

[CR64] McCormick, Susan M, Stacie R. Frye, Suzanne G. Eskin, Christina L. Teng, Chiung‐Mei Lu, Christopher G. Russell, Krishnan K. Chittur, Larry V. McIntire (2003) Microarray analysis of shear stressed endothelial cells. Biorheology 40(1):5–11 12454381

[CR65] McDougall SR, Anderson ARA, Chaplain MAJ, Sherratt JA (2002). Mathematical modelling of flow through vascular networks: implications for tumour-induced angiogenesis and chemotherapy strategies. Bull Math Biol.

[CR66] McDougall SR, Anderson ARA, Chaplain MAJ (2006). Mathematical modelling of dynamic adaptive tumour-induced angiogenesis: Clinical implications and therapeutic targeting strategies. J Theor Biol.

[CR67] Milde F, Bergdorf M, Koumoutsakos P (2008). A hybrid model for three-dimensional simulations of sprouting angiogenesis. Biophys J.

[CR68] Mongiat M, Andreuzzi E, Tarticchio G, Paulitti A (2016). Extracellular matrix, a hard player in angiogenesis. Int J Mol Sci.

[CR69] Nekka F, Kyriacos S, Kerrigan C, Cartilier L (1996). A model of growing vascular structures. Bull Math Biol.

[CR70] Nivlouei SJ, Soltani M, Carvalho J, Travasso R, Salimpour MR, Shirani E (2021). Multiscale modeling of tumor growth and angiogenesis: evaluation of tumor-targeted therapy. PLoS Comput Biol.

[CR71] Owen MR, Alarcón T, Maini PK, Byrne HM (2009). Angiogenesis and vascular remodelling in normal and cancerous tissues. J Math Biol.

[CR72] Peirce SM, Van Gieson EJ, Skalak TC (2004). Multicellular simulation predicts microvascular patterning and in silico tissue assembly. FASEB J.

[CR73] Perfahl H (2011). Multiscale modelling of vascular tumour growth in 3D: the roles of domain size and boundary conditions. PLoS One.

[CR74] Potente M, Gerhardt H, Carmeliet P (2011). Basic and therapeutic aspects of angiogenesis. Cell.

[CR75] Reinhart-King CA (2013). Mechanical and chemical signaling in angiogenesis.

[CR76] Ribatti D, Crivellato E (2009). Immune cells and angiogenesis. J Cell Mol Med.

[CR77] Risau W (1997). Mechanisms of angiogenesis. Nature.

[CR78] Rolland Y, Bézy-Wendling J, Duvauferrier R, Bruno A (1999). Modeling of the parenchymous vascularization and perfusion. Invest Radiol.

[CR79] Roman BL, Pekkan K (2012). Mechanotransduction in embryonic vascular development. Biomech Model Mechanobiol.

[CR80] Rouwkema J, Khademhosseini A (2016). Vascularization and angiogenesis in tissue engineering: beyond creating static networks. Trends Biotechnol.

[CR81] Sandau K, Kurz H (1994). Modelling of vascular growth processes: a stochastic biophysical approach to embryonic angiogenesis. J Microsc.

[CR82] Santos-Oliveira P (2015). The force at the tip - modelling tension and proliferation in sprouting angiogenesis. PLoS Comput Biol.

[CR83] Sato TN (2013). Mechanical and chemical regulation of arterial and venous specification. Stud Mechanobiol Tissue Eng Biomater.

[CR84] Schugart RC, Friedman A, Zhao R, Sen CK (2008). Wound angiogenesis as a function of tissue oxygen tension: a mathematical model. Proc Natl Acad Sci U S A.

[CR85] Scianna M, Bell CG, Preziosi L (2013). A review of mathematical models for the formation of vascular networks. J Theor Biol.

[CR86] Sharifpanah F, Behr S, Wartenberg M, Sauer H (2016). Mechanical strain stimulates vasculogenesis and expression of angiogenesis guidance molecules of embryonic stem cells through elevation of intracellular calcium, reactive oxygen species and nitric oxide generation. Biochim Biophys Acta - Mol Cell Res.

[CR87] Shibuya M (2011). Vascular endothelial growth factor (VEGF) and its receptor (VEGFR) signaling in angiogenesis: a crucial target for anti- and pro-angiogenic therapies. Genes Cancer.

[CR88] Shiu YT, Weiss JA, Hoying JB, Iwamoto MN, Joung IS, Quam CT (2005). The role of mechanical stresses in angiogenesis. Crit Rev Biomed Eng.

[CR89] Sieminski AL, Hebbel RP, Gooch KJ (2004). The relative magnitudes of endothelial force generation and matrix stiffness modulate capillary morphogenesis in vitro. Exp Cell Res.

[CR90] Stapor PC, Wang W, Murfee WL, Khismatullin DB (2011). The distribution of fluid shear stresses in capillary sprouts. Cardiovasc Eng Technol.

[CR91] Staton CA, Reed MWR, Brown NJ (2009). A critical analysis of current in vitro and in vivo angiogenesis assays. Int J Exp Pathol.

[CR92] Stepanova D, Byrne HM, Maini PK, Alarcón T (2021). A multiscale model of complex endothelial cell dynamics in early angiogenesis. PLoS Comput Biol.

[CR93] Stéphanou A, Le Floch S, Chauvière A (2015). A hybrid model to test the importance of mechanical cues driving cell migration in angiogenesis. Math Modell Nat Phenom.

[CR94] Stockmann C, Schadendorf D, Klose R, Helfrich I (2014). The impact of the immune system on tumor: angiogenesis and vascular remodeling. Front Oncol.

[CR95] Stokes CL, Lauffenburger DA, Williams SK (1991). Migration of individual microvessel endothelial cells: stochastic model and parameter measurement. J Cell Sci.

[CR96] Thi M-UD, Trocmé C, Montmasson M-P, Fanchon E, Toussaint B, Tracqui P (2012). Investigating metalloproteinases MMP-2 and MMP-9 mechanosensitivity to feedback loops involved in the regulation of in vitro angiogenesis by endogenous mechanical stresses. Acta Biotheoret.

[CR97] Ueda A, Koga M, Ikeda M, Kudo S, Tanishita K (2004). Effect of shear stress on microvessel network formation of endothelial cells with in vitro three-dimensional model. Am J Physiol - Hear Circ Physiol.

[CR98] Valastyan S, Weinberg RA (2011). Tumor metastasis: molecular insights and evolving paradigms. Cell.

[CR99] Vega R, Carretero M, Travasso RDM, Bonilla LL (2020). Notch signaling and taxis mechanims regulate early stage angiogenesis: a mathematical and computational model. PLoS Comput Biol.

[CR100] Vernon RB, Sage EH (1999). A novel, quantitative model for study of endothelial cell migration and sprout formation within three-dimensional collagen matrices. Microvasc Res.

[CR101] Vernon RB, Angello JC, Iruela-Arispe L, Lane TF, Sage EH (1992). Reorganization of basement membrane matrices by cellular traction promotes the formation of cellular networks in vitro. Lab Investig.

[CR102] Vernon RB, Lara SL, Drake CJ, Luisa Iruela-Arispe M, Angello JC, Little CD, Wight TN, Helene Sage E (1995). Organized type I collagen influences endothelial patterns during “spontaneous angiogenesis in vitro”: planar cultures as models of vascular development. In Vitro Cell Dev Biol Animal.

[CR103] Vilanova G, Colominas I, Gomez H (2014). Coupling of discrete random walks and continuous modeling for three-dimensional tumor-induced angiogenesis. Comput Mech.

[CR104] von Rüden C, Augat P, Hollensteiner M (2021). The role of mechanical stimulation in the enhancement of bone healing. Injury.

[CR105] Wang S, Sun J, Xiao Y, Lu Y, Zhang DD, Wong PK (2017). Intercellular tension negatively regulates angiogenic sprouting of endothelial tip cells via notch1-Dll4 signaling. Adv Biosyst.

[CR110] Wood Levi, Kamm Roger, Asada Harry (2011). Stochastic modeling and identification of emergent behaviors of an Endothelial Cell population in angiogenic pattern formation. Int J Robot Res.

[CR106] Wu M, Frieboes HB, McDougall SR, Chaplain MAJ, Cristini V, Lowengrub J (2013). The effect of interstitial pressure on tumor growth: coupling with the blood and lymphatic vascular systems. J Theor Biol.

[CR107] Yu CY, Chae J, Buehler MJ, Hunter CP, Mooney DJ (2009). Cyclic tensile strain triggers a sequence of autocrine and paracrine signaling to regulate angiogenic sprouting in human vascular cells. Proc Natl Acad Sci USA.

[CR108] Zeiger AS (2016). Static mechanical strain induces capillary endothelial cell cycle re-entry and sprouting. Phys Biol.

[CR109] Zheng X, Wise SM, Cristini V (2005). Nonlinear simulation of tumor necrosis, neo-vascularization and tissue invasion via an adaptive finite-element/level-set method. Bull Math Biol.

